# Supplementary figures and images for: The impact of race and ethnicity on outcomes in 19,584 adults hospitalized with COVID-19
Source: PLoS One. 2021 Jul 21;16(7):e0254809. doi: 10.1371/journal.pone.0254809 (PMC8294547; doi:10.1371/journal.pone.0254809)

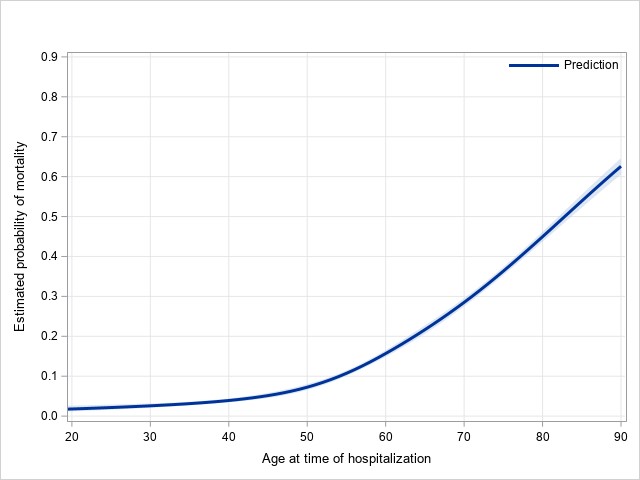

Supplement: S1 Fig — (JPG) [file pone.0254809.s001.jpg]

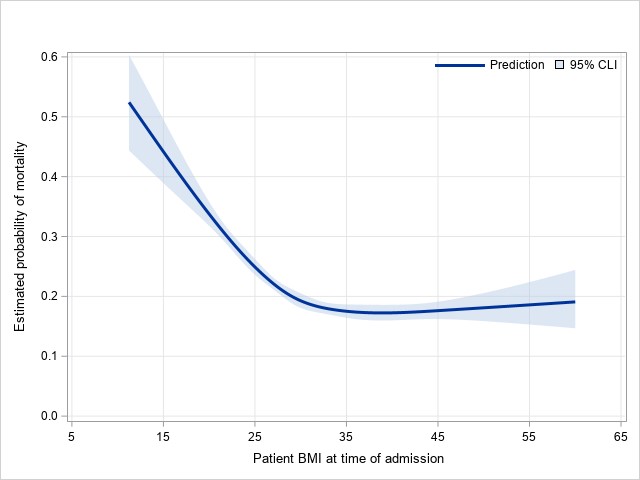

Supplement: S2 Fig — (JPG) [file pone.0254809.s002.jpg]

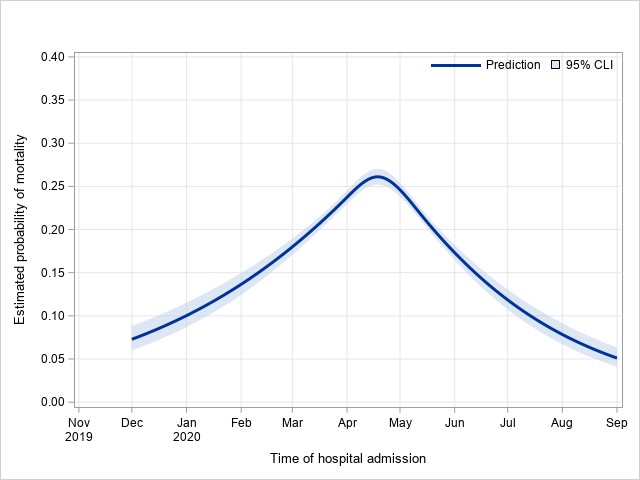

Supplement: S3 Fig — (JPG) [file pone.0254809.s003.jpg]
