# Supplementary material for: The impact of race and ethnicity on outcomes in 19,584 adults hospitalized with COVID-19
Source: PLoS One. 2021 Jul 21;16(7):e0254809. doi: 10.1371/journal.pone.0254809 (PMC8294547; doi:10.1371/journal.pone.0254809)
Supplement: S1 Table — (PDF) [file pone.0254809.s004.pdf]

COVID-19 Clinical Diagnosis		
ICD-10-CM	A41.89	Other specified sepsis
ICD-10-CM	B34.2	Coronavirus infection, unspecified
ICD-10-CM	B97.21	SARS-associated coronavirus as the cause of diseases classified elsewhere
ICD-10-CM	B97.29	Other coronavirus as the cause of diseases classified elsewhere
ICD-10-CM	J12.81	Pneumonia due to SARS-associated coronavirus
ICD-10-CM	J12.89	Other viral pneumonia
ICD-10-CM	U07.1	COVID-19, virus identified
ICD-10-WHO	U07.2	COVID-19, virus not identified
