# Supplementary material for: The impact of race and ethnicity on outcomes in 19,584 adults hospitalized with COVID-19
Source: PLoS One. 2021 Jul 21;16(7):e0254809. doi: 10.1371/journal.pone.0254809 (PMC8294547; doi:10.1371/journal.pone.0254809)
Supplement: S2 Table — (PDF) [file pone.0254809.s005.pdf]

	Known Status	Transferred	Status Unknown	Total
**American Indian or Alaska**	474	94	20	588
	80.6%	16.0%	3.4%	
**Asian or Pacific islander**	679	142	47	868
	78.2%	16.4%	5.4%	
**Black or African American**	4215	1020	696	5931
	71.1%	17.2%	11.7%	
**White**	9994	2789	662	13445
	74.3%	20.7%	4.9%	
**Total**	15362	4045	1425	20832
