# Supplementary material for: The impact of race and ethnicity on outcomes in 19,584 adults hospitalized with COVID-19
Source: PLoS One. 2021 Jul 21;16(7):e0254809. doi: 10.1371/journal.pone.0254809 (PMC8294547; doi:10.1371/journal.pone.0254809)
Supplement: S3 Table — (PDF) [file pone.0254809.s006.pdf]

		Model 1 Excluding Comorbidities and BMI* (n=19,537)		Model 2 Including Comorbidities and BMI** (n=11,045)	
		OR	95% CI	OR	95% CI
Sex	Male vs Female	1.53	(1.41-1.66)	1.46	(1.31-1.62)
Race (Ref:White)	American Indian or Alaska Native	1.64	(1.21-2.23)	1.21	(0.80-1.83)
	Asian or Pacific Islander	0.78	(0.63-0.98)	0.78	(0.57-1.08)
	Black or African American	1.13	(1.01-1.27)	1.07	(0.93-1.23)
	Mixed racial group	0.7	(0.07-8.07)	1.99	(0.14-27.94)
	Other racial group	0.96	(0.83-1.11)	1.03	(0.84-1.27)
	Unknown racial group	1.7	(1.39-2.01)	1.58	(1.20-2.07)
Ethnicity (Ref: non-Hispanic)	Ethnic group unknown	1.00	(0.87-1.16)	1.04	(0.83-1.29)
	Hispanic or Latino	0.74	(0.65-0.83)	0.71	(0.59-0.86)
Comorbidities	Asthma			0.73	(0.60-0.88)
	Diabetes			1.27	(1.13-1.42)
	Heart failure			1.29	(1.13-1.47)
	CKD			1.50	(1.32-1.69)

*Both models include adjustment for age, ZIP code, and admission date

**Comorbidity and BMI model includes adjustment factors in model 1 as well as for BMI, modeled as nonlinear using restricted cubic spline
