# Supplementary material for: The impact of race and ethnicity on outcomes in 19,584 adults hospitalized with COVID-19
Source: PLoS One. 2021 Jul 21;16(7):e0254809. doi: 10.1371/journal.pone.0254809 (PMC8294547; doi:10.1371/journal.pone.0254809)
Supplement: Attachment — Submitted filename: Reviewer comment_PONE-D-21-07038_14Apr2021.docx [file pone.0254809.s007.docx]

**Reviewer comment for PLOS ONE**

Date of review: 14 April 2021

Manuscript Number: PONE-D-21-07038

Article Type: Research Article

Full Title: The Impact of Race and Ethnicity on Outcomes in 19,584 adults Hospitalized with COVID-19

Suggested title: The Impact of Race and Ethnicity on Outcomes in adults Hospitalized with COVID-19 in the United States

Corresponding Author: Ann Marie Navar, UT Southwestern: The University of Texas Southwestern Medical Center, Dallas, UNITED STATES

**Reviewer comment**

Summary of the research and overall impression

This manuscript identified whether race and ethnicity impact on risk of death among those hospitalized for COVID-19 in the US. Generally, the paper presented well. The introduction is short and required to add more data about the burden of the disease and background situation in the hospitals or States included in the study. Methods session presents how the analysis was carried out. Results are comprehensive, however, a few editions are suggested.

*BTW, I don’t see the line number in the manuscript in PDF version and it is difficult to provide the direction of the comments.*

Discussion on specific area of improvement

Abstract

Well-written and clearly presented abstract.

1. Results - line 3: “[22.7% vs 20.8%, unadjusted 1.12 (95% CI 1.02-. I think it should be unadjusted **OR** 1.12.
2. The paper was said “in adult”, please add the mean (or median) age of participants in the results.

Introduction

3. Introduction is too short; it is incomplete information. Suggest to add more data on COVID-19 pandemic outbreak prevalence of (burden) across the US, differences in States, proportion of hospitalized patient and mortality rates, etc. (burden of disease)
4. Suggest to include other studies findings on race and ethnicity diversion on disease burden and mortality in other infectious diseases and any literature on COVID-19 in other countries. No data is the data to present. (gap in knowledge)
5. Suggest to add how race and ethnicity can influence for diagnosis and treatment for preventing life-threatening situation or reduce the mortality in certain diseases, more appropriately with infectious diseases or COVID-19. (gap in knowledge)
6. Lastly, why this study is important to conduct (justification) and how this study can provide information or input to the health system.

Materials and methods

7. Pg. 10, 3^rd^ paragraph, 1^st^ line: “ICD-10 diagnosis codes” – please spell this out and add reference.
8. Pg. 10, 3^rd^ paragraph, 3^rd^ line: “Cerner EHR” – please spell this out. Add reference for using cut off point for obesity.

Results

9. Pg. 12, 1^st^ paragraph: to add text about age (said “younger”, but please specify), sex and geographic distribution of cases
10. Pg. 12, the title is not matched with the text, it is talking about “demographic characteristics and comorbidity of the cases”, not “race and ethnicity”
11. Again, the paragraph does not refer to any table. Please add reference table for the data presented in the text.
12. Tables: the data (numbers) is suggested to right-aligned, rather than centered.
13. Pg. 18, table 3: Please add odds ratios (OR) and CI in the tables. You may use * for p-values that are significant (eg. *p<0.001, ^#^p<0.01)

Discussion and conclusions

14. Pg. 19, 1^st^ paragraph, line 1: please use the exact sample size, do not use “nearly 20,000 patients” and use “52 health systems”.
15. Pg. 20, 2^nd^ paragraph: line 1-3: please add reference.

Recommendation

I would like to propose the **minor revision** for this manuscript.
