# Supplementary material for: The impact of race and ethnicity on outcomes in 19,584 adults hospitalized with COVID-19
Source: PLoS One. 2021 Jul 21;16(7):e0254809. doi: 10.1371/journal.pone.0254809 (PMC8294547; doi:10.1371/journal.pone.0254809)
Supplement: Attachment — Submitted filename: PLOS one responses to reviewers_5.13.docx [file pone.0254809.s008.docx]

Journal Requirements:
Please review your reference list to ensure that it is complete and correct. If you have cited papers that have been retracted, please include the rationale for doing so in the manuscript text, or remove these references and replace them with relevant current references. Any changes to the reference list should be mentioned in the rebuttal letter that accompanies your revised manuscript. If you need to cite a retracted article, indicate the article’s retracted status in the References list and also include a citation and full reference for the retraction notice.

When submitting your revision, we need you to address these additional requirements.

1. Please ensure that your manuscript meets PLOS ONE's style requirements, including those for file naming. The PLOS ONE style templates can be found at

[https://journals.plos.org/plosone/s/file?id=wjVg/PLOSOne_formatting_sample_main_body.pdf](https://nam10.safelinks.protection.outlook.com/?url=https%3A%2F%2Fjournals.plos.org%2Fplosone%2Fs%2Ffile%3Fid%3DwjVg%2FPLOSOne_formatting_sample_main_body.pdf&data=04%7C01%7CChristy.Dueck%40Cerner.com%7C1087f2ed0d13420d44f808d905c5c489%7Cfbc493a80d244454a815f4ca58e8c09d%7C0%7C0%7C637547169375084364%7CUnknown%7CTWFpbGZsb3d8eyJWIjoiMC4wLjAwMDAiLCJQIjoiV2luMzIiLCJBTiI6Ik1haWwiLCJXVCI6Mn0%3D%7C1000&sdata=HpHgbwnTREs7XmzojjSa0mOp3R0lE0U5O7cocfIRP4A%3D&reserved=0) and

[https://journals.plos.org/plosone/s/file?id=ba62/PLOSOne_formatting_sample_title_authors_affiliations.pdf](about:blank)

**The manuscript has been reformatted as requested to align with guidelines.**

2. Thank you for providing the following Funding Statement:

"This study was supported by Cerner Corporation who provided access to the dataset and employee time to participate on the research study. The dataset for this study was created by Cerner for use by academic researchers with support from Amazon Web Services, and is available for access to external researchers upon request. Ann Marie Navar and Eric Peterson receive support for consulting to Cerner Corporation for activities outside of this work, but were not compensated for work on this analysis. Rob Taylor, Qingjiang Hou, and Stacey Purinton are employees of Cerner Corporation. They were not compensated for their work on this study beyond their regular salary for employment with Cerner."

 We note that one or more of the authors is affiliated with the funding organization, indicating the funder may have had some role in the design, data collection, analysis or preparation of your manuscript for publication; in other words, the funder played an indirect role through the participation of the co-authors.

If the funding organization did not play a role in the study design, data collection and analysis, decision to publish, or preparation of the manuscript and only provided financial support in the form of authors' salaries and/or research materials, please review your statements relating to the author contributions, and ensure you have specifically and accurately indicated the role(s) that these authors had in your study in the Author Contributions section of the online submission form. Please make any necessary amendments directly within this section of the online submission form.  Please also update your Funding Statement to include the following statement: “The funder provided support in the form of salaries for authors [insert relevant initials], but did not have any additional role in the study design, data collection and analysis, decision to publish, or preparation of the manuscript. The specific roles of these authors are articulated in the ‘author contributions’ section.”

If the funding organization did have an additional role, please state and explain that role within your Funding Statement.

Please also provide an updated Competing Interests Statement declaring this commercial affiliation along with any other relevant declarations relating to employment, consultancy, patents, products in development, or marketed products, etc.

Within your Competing Interests Statement, please confirm that this commercial affiliation does not alter your adherence to all PLOS ONE policies on sharing data and materials by including the following statement: "This does not alter our adherence to  PLOS ONE policies on sharing data and materials.” (as detailed online in our guide for authors [http://journals.plos.org/plosone/s/competing-interests](about:blank)). If this adherence statement is not accurate and  there are restrictions on sharing of data and/or materials, please state these. Please note that we cannot proceed with consideration of your article until this information has been declared.

**Cerner was not a “funder” in the sense that funds were made available to the project, which is why we didn’t include them initially. This has been added and that box has checked. We took this information out of the “acknowledgement section” per formatting requirements. A funding statement is now in the comments section of the submission system:**

Cerner provided in-kind support for the study by providing access to data and through the work of Cerner co-authors. Cerner co-authors would have received their regular salary during the course of their work on this project, but this was not linked directly to this manuscript

Please know it is PLOS ONE policy for corresponding authors to declare, on behalf of all authors, all potential competing interests for the purposes of transparency. PLOS defines a competing interest as anything that interferes with, or could reasonably be perceived as interfering with, the full and objective presentation, peer review, editorial decision-making, or publication of research or non-research articles submitted to one of the journals. Competing interests can be financial or non-financial, professional, or personal. Competing interests can arise in relationship to an organization or another person. Please follow this link to our website for more details on competing interests: [http://journals.plos.org/plosone/s/competing-interests](about:blank)

**The competing interests statement is now in the comments section of the submission system, and author contributions are clearly defined in the manuscript:**

**The updated financial statement provided outlines this information now in more detail:**

Cerner Corporation-employed co-authors (QH, RT, SP) received salary from Cerner corporation during the time they participated on this research project. Cerner Corp. provided in-kind support for the study through their effort and through providing access to data. Cerner Corp. co-authors were a part of the study team and had roles in creating the dataset and data acquisition (RT, SP), data analysis (QH), the concept and design of the study, data interpretation, and critical revision of the manuscript (QH, RT, SP). EP and AMN receive fees for research consulting to Cerner Corporation outside of the present work. EP and AMN were not compensated for their work on this manuscript.

3. We note that you have indicated that data from this study are available upon request. PLOS only allows data to be available upon request if there are legal or ethical restrictions on sharing data publicly. For information on unacceptable data access restrictions, please see [http://journals.plos.org/plosone/s/data-availability#loc-unacceptable-data-access-restrictions](https://nam10.safelinks.protection.outlook.com/?url=http%3A%2F%2Fjournals.plos.org%2Fplosone%2Fs%2Fdata-availability%23loc-unacceptable-data-access-restrictions&data=04%7C01%7CChristy.Dueck%40Cerner.com%7C1087f2ed0d13420d44f808d905c5c489%7Cfbc493a80d244454a815f4ca58e8c09d%7C0%7C0%7C637547169375084364%7CUnknown%7CTWFpbGZsb3d8eyJWIjoiMC4wLjAwMDAiLCJQIjoiV2luMzIiLCJBTiI6Ik1haWwiLCJXVCI6Mn0%3D%7C1000&sdata=tFbBMukiLwoUgqyiUAB3cMrn5sQx3B7LVGngWSIQcKo%3D&reserved=0).

In your revised cover letter, please address the following prompts:

a) If there are ethical or legal restrictions on sharing a de-identified data set, please explain them in detail (e.g., data contain potentially identifying or sensitive patient information) and who has imposed them (e.g., an ethics committee). Please also provide contact information for a data access committee, ethics committee, or other institutional body to which data requests may be sent.

**Thank you for the opportunity to clarify. We have added this information to the cover letter:**

Data from the Cerner Real World Data COVID dataset are available on request to Cerner corporation [Kendra.Stillwell@Cerner.com](mailto:Kendra.Stillwell@Cerner.com). Per the user agreements between Cerner and the contributing health systems, all research requests must be approved by the governance council which consists of representatives from both Cerner and contributing health systems. Upon approval, researchers can access the de-identified datasets on the Cerner platform; individual patient data (even de-identified data) are never released outside of the analytic environment to protect patient confidentiality based on the agreements in place between Cerner and participating sites.

**This has also been clarified in the manuscript.**

Per the user agreements between Cerner and participating health systems, individual patient data cannot be shared externally but approved researchers can access the data for individual studies.

b) If there are no restrictions, please upload the minimal anonymized data set necessary to replicate your study findings as either Supporting Information files or to a stable, public repository and provide us with the relevant URLs, DOIs, or accession numbers. Please see [http://www.bmj.com/content/340/bmj.c181.long](https://nam10.safelinks.protection.outlook.com/?url=http%3A%2F%2Fwww.bmj.com%2Fcontent%2F340%2Fbmj.c181.long&data=04%7C01%7CChristy.Dueck%40Cerner.com%7C1087f2ed0d13420d44f808d905c5c489%7Cfbc493a80d244454a815f4ca58e8c09d%7C0%7C0%7C637547169375094368%7CUnknown%7CTWFpbGZsb3d8eyJWIjoiMC4wLjAwMDAiLCJQIjoiV2luMzIiLCJBTiI6Ik1haWwiLCJXVCI6Mn0%3D%7C1000&sdata=VEmteDhai9GIRCeIDYNpI9MTWNXOToN286cb8NU22HU%3D&reserved=0) for guidelines on how to de-identify and prepare clinical data for publication. For a list of acceptable repositories, please see [http://journals.plos.org/plosone/s/data-availability#loc-recommended-repositories](https://nam10.safelinks.protection.outlook.com/?url=http%3A%2F%2Fjournals.plos.org%2Fplosone%2Fs%2Fdata-availability%23loc-recommended-repositories&data=04%7C01%7CChristy.Dueck%40Cerner.com%7C1087f2ed0d13420d44f808d905c5c489%7Cfbc493a80d244454a815f4ca58e8c09d%7C0%7C0%7C637547169375094368%7CUnknown%7CTWFpbGZsb3d8eyJWIjoiMC4wLjAwMDAiLCJQIjoiV2luMzIiLCJBTiI6Ik1haWwiLCJXVCI6Mn0%3D%7C1000&sdata=AtNQ%2B5b7u1feacOG9fzteh7mlxxoyECuII06TrsZgFM%3D&reserved=0).

We will update your Data Availability statement on your behalf to reflect the information you provide.

**See above for details about data access.**

4. Please include captions for your Supporting Information files at the end of your manuscript, and update any in-text citations to match accordingly. Please see our Supporting Information guidelines for more information: [http://journals.plos.org/plosone/s/supporting-information](about:blank).

**The supplement has been edited to include a figure captain for all captions.**

[Note: HTML markup is below. Please do not edit.]

Reviewers' comments:

Reviewer's Responses to Questions

**Comments to the Author**

1. Is the manuscript technically sound, and do the data support the conclusions?

The manuscript must describe a technically sound piece of scientific research with data that supports the conclusions. Experiments must have been conducted rigorously, with appropriate controls, replication, and sample sizes. The conclusions must be drawn appropriately based on the data presented.

Reviewer #1: Yes

Reviewer #2: Yes

2. Has the statistical analysis been performed appropriately and rigorously?

Reviewer #1: Yes

Reviewer #2: I Don't Know

3. Have the authors made all data underlying the findings in their manuscript fully available?

The [PLOS Data policy](https://nam10.safelinks.protection.outlook.com/?url=http%3A%2F%2Fwww.plosone.org%2Fstatic%2Fpolicies.action%23sharing&data=04%7C01%7CChristy.Dueck%40Cerner.com%7C1087f2ed0d13420d44f808d905c5c489%7Cfbc493a80d244454a815f4ca58e8c09d%7C0%7C0%7C637547169375104362%7CUnknown%7CTWFpbGZsb3d8eyJWIjoiMC4wLjAwMDAiLCJQIjoiV2luMzIiLCJBTiI6Ik1haWwiLCJXVCI6Mn0%3D%7C1000&sdata=9cQN%2B5uRnIcKMOxNanQkBGDbIIJnAASeyUo4Sjp5nz8%3D&reserved=0) requires authors to make all data underlying the findings described in their manuscript fully available without restriction, with rare exception (please refer to the Data Availability Statement in the manuscript PDF file). The data should be provided as part of the manuscript or its supporting information, or deposited to a public repository. For example, in addition to summary statistics, the data points behind means, medians and variance measures should be available. If there are restrictions on publicly sharing data—e.g. participant privacy or use of data from a third party—those must be specified.

Reviewer #1: No

Reviewer #2: Yes

**See above for edits on the data sharing statements**

4. Is the manuscript presented in an intelligible fashion and written in standard English?

PLOS ONE does not copyedit accepted manuscripts, so the language in submitted articles must be clear, correct, and unambiguous. Any typographical or grammatical errors should be corrected at revision, so please note any specific errors here.

Reviewer #1: Yes

Reviewer #2: Yes

5. Review Comments to the Author

Please use the space provided to explain your answers to the questions above. You may also include additional comments for the author, including concerns about dual publication, research ethics, or publication ethics. (Please upload your review as an attachment if it exceeds 20,000 characters)

Reviewer #1: Reviewer comment for PLOS ONE
Date of review: 14 April 2021
Manuscript Number: PONE-D-21-07038
Article Type: Research Article
Full Title: The Impact of Race and Ethnicity on Outcomes in 19,584 adults Hospitalized with COVID-19
Suggested title: The Impact of Race and Ethnicity on Outcomes in 19,584 adults Hospitalized with COVID-19 in the United States
Corresponding Author: Ann Marie Navar, UT Southwestern: The University of Texas Southwestern Medical Center, Dallas, UNITED STATES

Reviewer comment
Summary of the research and overall impression
This manuscript identified whether race and ethnicity impact on risk of death among those hospitalized for COVID-19 in the US. Generally, the paper presented well. The introduction is short and required to add more data about the burden of the disease and background situation in the hospitals or States included in the study. Methods session presents how the analysis was carried out. Results are comprehensive, however, a few editions are suggested.
BTW, I don’t see the line number in the manuscript in PDF version and it is difficult to provide the direction of the comments.
Discussion on specific area of improvement
Abstract
Well-written and clearly presented abstract.
1. Results - line 3: “[22.7% vs 20.8%, unadjusted 1.12 (95% CI 1.02-. I think it should be unadjusted OR 1.12.

**This has been corrected – thank you**

2. The paper was said “in adult”, please add the mean (or median) age of participants in the results.

**This has been added as requested**

*Through August 2020, 19,584 patients with median age 52 years were hospitalized with COVID-19, including n=4,215 (21.5%) Black and n=5,761 (29.4%) Hispanic persons*

Introduction
3. Introduction is too short; it is incomplete information. Suggest to add more data on COVID-19 pandemic outbreak prevalence of (burden) across the US, differences in States, proportion of hospitalized patient and mortality rates, etc. (burden of disease)

**We added data on the burden of disease in COVID-19 to the first paragraph of the intro**

*The COVID-19 pandemic caused approximately 375,000 deaths in the United States in 2020, and was either the cause of death or contributing cause of death for 11.3% of all deaths in the United States.[ ] Likely due to COVID-19, the age-adjusted mortality increased in the United States by 15.9% in 2020.[ ] Among patients hospitalized for COVID-19, the mortality rate has generally decreased over time, but has been shown to vary across hospitals, with one study showing a 50% variability in risk-standardized event rates of mortality or referral to hospice among COVID-19 patients in the first six months of the pandemic.[ ]*

4. Suggest to include other studies findings on race and ethnicity diversion on disease burden and mortality in other infectious diseases and any literature on COVID-19 in other countries. No data is the data to present. (gap in knowledge)

**As this study is focused on the US we did not include race/ethnicity data on COVID-19 from other countries.**

5. Suggest to add how race and ethnicity can influence for diagnosis and treatment for preventing life-threatening situation or reduce the mortality in certain diseases, more appropriately with infectious diseases or COVID-19. (gap in knowledge)

**We added the following to the introduction**

*Accurate information regarding the impact of race and ethnicity on outcomes is important to help physicians identify higher risk patients when admitted, to identify potential biological mechanisms affecting prognosis, and to target public health interventions appropriately. Most importantly, differences in outcomes by race and ethnicity may serve as an indicator of systemic biases in the healthcare system, with differential treatment leading to differential outcomes.*

6. Lastly, why this study is important to conduct (justification) and how this study can provide information or input to the health system.

**See response to the above comment which we feel highlights the importance of understanding variability by race**

Materials and methods
7. Pg. 10, 3rd paragraph, 1st line: “ICD-10 diagnosis codes” – please spell this out and add reference.

**This has been spelled out. We noted an error in that we also used MEDCIN and SNOMED codes, this has been clarified in the manuscript. We don’t have a specific reference for ICD-10.**

8. Pg. 10, 3rd paragraph, 3rd line: “Cerner EHR” – please spell this out. Add reference for using cut off point for obesity.

**EHR has been fully spelled out. This is a universally used cutoff for BMI for obesity.**

Results
9. Pg. 12, 1st paragraph: to add text about age (said “younger”, but please specify), sex and geographic distribution of cases

**We added the numbers into the text for this section as requested.**

*Overall, 51.0% (n=9994) of our sample was white while 21.5% were recorded as Black (n=4215). Table 1 shows differences between Black and white adults admitted with COVID-19. Black adults were younger (median age 59 vs 62 years), less likely to be male (47.3% vs 53.0%), less likely to be Hispanic (1.9% vs 36.3%), had higher BMIs (median BMI 30.7 vs 38.9), and different distribution of insurance coverage (p<0.001 for all). Black patients also had higher rates of diabetes, hypertension, coronary artery disease, heart failure, chronic kidney disease, and end stage renal disease, and lower rates of COPD and asthma (see Table 1).*

10. Pg. 12, the title is not matched with the text, it is talking about “demographic characteristics and comorbidity of the cases”, not “race and ethnicity”

**On page 12 we are discussing characteristics of patients by race and ethnicity. We discuss differences in comorbidities and demographics of cases by race and ethnicity, which is why this appears in this section.**

11. Again, the paragraph does not refer to any table. Please add reference table for the data presented in the text.

**We have clarified this-**

*Of the 19,584 patients included, n=4050 (20.7%) died during the hospital stay ~~(Table 2)~~.* *Table 2 shows characteristics of adults overall and stratified by in-hospital mortality.*

*12. Tabl*es: the data (numbers) is suggested to right-aligned, rather than centered.

**We will defer to the editors on the journal preference regarding formatting and choice of left, right, vs center aligned.**

13. Pg. 18, table 3: Please add odds ratios (OR) and CI in the tables. You may use * for p-values that are significant (eg. *p<0.001, #p<0.01)

**We have added these ORs to this table.**

Discussion and conclusions
14. Pg. 19, 1st paragraph, line 1: please use the exact sample size, do not use “nearly 20,000 patients” and use “52 health systems”.

**This has been corrected as follows:**

*In a nationwide, EHR-based database of 19,584 patients from 52 health systems across the United States*

15. Pg. 20, 2nd paragraph: line 1-3: please add reference.

**This intro sentence sets up the paragraph where we provide specific references.**

Recommendation
I would like to propose the minor revision for this manuscript.

**This has been added** (Pro00105396)

Please add the ethical approval number from Duke University.

**Thank you to the reviewer for the thoughtful and thorough review.**

Reviewer #2: The investigators conducted a retrospective cohort study to evaluate, among patients hospitalized for COVID-19, the differences in race and ethnicity and their association with death.
The manuscript is presented clearly and concisely. The following comments may help in improving the manuscript.

General
1. Persons/patients, consider using one uniformly.

**Except when discussing national data (and referring specifically to populations, not patients), we have changed “persons” to “patients” throughout**

2. Mortality/death consider using one uniformly.

**We have changed “death” to “mortality” throughout**

3. clinical comorbidities/comorbidities consider using one uniformly.

**We have simplified and use “comorbidities” throughout**

Methods
1. “..we utilized any available encounter within 3 years of and including the analysis hospitalization”. Consider revising this text for clarity.

**This has been clarified**

*To determine race and ethnicity, we evaluated for the presence of an indicator of race within 3 years of and including the analysis hospitalization.*

2. “those discharged on hospice”. Should it be“to” instead of “on”?

**Thank you- this is corrected**

Results
1. Consider reporting, % of participants for whom data on race and ethnicity were missing.

**This has been added to the first paragraph**

*Race data was missing for n=1,017 (5.19%) of patients overall.*

2. “Compared with White adults, black adults had similar rates of myocardial infarction, but higher rates of myocardial infarction, stroke, ventricular tachycardia, and pulmonary embolism.” In this sentence, should the text “but higher rates of myocardial infarction” be omitted?

**Thank you- this was an error in the text and has been corrected.**

*Compared with White adults, Black adults had similar rates of myocardial infarction, but higher rates of ~~myocardial infarction,~~ stroke, ventricular tachycardia, and pulmonary embolism*.

Tables
1. Expand abbreviations in footnotes (e.g. ESRD)

**Thank you- these have been added**

2. There is no data in Table 1 on complications, and survival, consider revising the Table title

**Thank you for this- we have corrected the title**

***Table 1: Characteristics of white, Black, Hispanic, and non-Hispanic patients hospitalized with COVID-19***

Discussion
1. Outcome (mortality) data for a significant proportion of participants (about 31%) was not available. Consider mentioning this as a limitation and its likely implications.

**This statement is based on our note that 6.1% were still hospitalized, 1.6% went to hospice, 16.6% were transferred, and discharge status was unknown for 6.5%. We now break this down by race as well in the supplement, and discuss this in the limitations. The overall difference between Black and White patients who were transferred was 3.3%, which is unlikely to impact our findings. Similarly, while there was a higher rate of unknown discharge status in in Black / African American patients, survival would have to have been substantially different in this population to impact findings.**

*If transfers were variable by race, and survival was different among those who were transferred vs remained hospitalized, this may have impacted our findings, which may only apply to those patients who are not transferred.*

Conclusion
1. Consider revising to exclude redundant text and for clarity.

**We have made revisions throughout the conclusion to help tighten up the messaging as requested.**

6. PLOS authors have the option to publish the peer review history of their article ([what does this mean?](https://nam10.safelinks.protection.outlook.com/?url=https%3A%2F%2Fjournals.plos.org%2Fplosone%2Fs%2Feditorial-and-peer-review-process%23loc-peer-review-history&data=04%7C01%7CChristy.Dueck%40Cerner.com%7C1087f2ed0d13420d44f808d905c5c489%7Cfbc493a80d244454a815f4ca58e8c09d%7C0%7C0%7C637547169375104362%7CUnknown%7CTWFpbGZsb3d8eyJWIjoiMC4wLjAwMDAiLCJQIjoiV2luMzIiLCJBTiI6Ik1haWwiLCJXVCI6Mn0%3D%7C1000&sdata=OMqJhCjEkYxQZtXf43TzqX%2BouSlpnjByqQzhM%2BagqHk%3D&reserved=0)). If published, this will include your full peer review and any attached files.


If you choose “no”, your identity will remain anonymous but your review may still be made public.


**Do you want your identity to be public for this peer review?** For information about this choice, including consent withdrawal, please see our [Privacy Policy](https://nam10.safelinks.protection.outlook.com/?url=https%3A%2F%2Fwww.plos.org%2Fprivacy-policy&data=04%7C01%7CChristy.Dueck%40Cerner.com%7C1087f2ed0d13420d44f808d905c5c489%7Cfbc493a80d244454a815f4ca58e8c09d%7C0%7C0%7C637547169375114360%7CUnknown%7CTWFpbGZsb3d8eyJWIjoiMC4wLjAwMDAiLCJQIjoiV2luMzIiLCJBTiI6Ik1haWwiLCJXVCI6Mn0%3D%7C1000&sdata=eI9xD7vSJic1OaYiJj%2Bp7nv17%2FBBRhM50QBRNrMgYdI%3D&reserved=0).

Reviewer #1: **Yes:** Poe Poe Aung

Reviewer #2: No

While revising your submission, please upload your figure files to the Preflight Analysis and Conversion Engine (PACE) digital diagnostic tool, [https://pacev2.apexcovantage.com/](https://nam10.safelinks.protection.outlook.com/?url=https%3A%2F%2Fpacev2.apexcovantage.com%2F&data=04%7C01%7CChristy.Dueck%40Cerner.com%7C1087f2ed0d13420d44f808d905c5c489%7Cfbc493a80d244454a815f4ca58e8c09d%7C0%7C0%7C637547169375114360%7CUnknown%7CTWFpbGZsb3d8eyJWIjoiMC4wLjAwMDAiLCJQIjoiV2luMzIiLCJBTiI6Ik1haWwiLCJXVCI6Mn0%3D%7C1000&sdata=a96oOTTKmDlJ2OCTl%2FhXE2J%2B23RCggxS7pNfHvTBnRo%3D&reserved=0). PACE helps ensure that figures meet PLOS requirements. To use PACE, you must first register as a user. Registration is free. Then, login and navigate to the UPLOAD tab, where you will find detailed instructions on how to use the tool. If you encounter any issues or have any questions when using PACE, please email PLOS at [figures@plos.org](mailto:figures@plos.org). Please note that Supporting Information files do not need this step.
