# Supplementary material for: The impact of race and ethnicity on outcomes in 19,584 adults hospitalized with COVID-19
Source: PLoS One. 2021 Jul 21;16(7):e0254809. doi: 10.1371/journal.pone.0254809 (PMC8294547; doi:10.1371/journal.pone.0254809)
Supplement: Attachment — Submitted filename: Reviewer comment_PONE-D-21-07038_R1_23June2021.docx [file pone.0254809.s009.docx]

**Reviewer comment for PLOS ONE**

Date of review: 23 June 2021

Manuscript Number: PONE-D-21-07038_R1

Article Type: Research Article

Full Title: The Impact of Race and Ethnicity on Outcomes in 19,584 adults Hospitalized with COVID-19

Corresponding Author: Ann Marie Navar, UT Southwestern: The University of Texas Southwestern Medical Center, Dallas, UNITED STATES

**Reviewer comment**

Summary of the research and overall impression

The manuscript was revised according to the reviewer’s comment, improved and ready for publication after revising the title by adding “in the United States”.

Discussion on specific area of improvement

Suggested title: The Impact of Race and Ethnicity on Outcomes in 19,584 adults Hospitalized with COVID-19 in the United States [to add the name of the country at the end of the title]

Recommendation

I would like to **accept** this manuscript.
